# 

**Published:** 1886-10-16

**Authors:** 


PRICE ONE PENNY.

No. 3, Vol. I.

October 16th, 1886.

Per Annum, 6s. 6d., post free.
Monthly Parts, 6d.; post free, 7½d.
Ditto, per Ann., 7s. 6d., post free.

